# STAT6 siRNA Matrix-Loaded Gelatin Nanocarriers: Formulation, Characterization, and *Ex Vivo* Proof of Concept Using Adenocarcinoma Cells

**DOI:** 10.1155/2013/858946

**Published:** 2013-09-26

**Authors:** Susanne R. Youngren, Rakesh K. Tekade, Brianne Gustilo, Peter R. Hoffmann, Mahavir B. Chougule

**Affiliations:** ^1^Department of Pharmaceutical Science, The Daniel K. Inouye College of Pharmacy, University of Hawai'i at Hilo, 200 W. Kawili Street, Hilo, HI 96720, USA; ^2^Department of Cell and Molecular Biology, John A. Burns School of Medicine, University of Hawai'i, 651 Ilalo Street, Honolulu, HI 96813, USA

## Abstract

The clinical utility of siRNA therapy has been hampered due to poor cell penetration, nonspecific effects, rapid degradation, and short half-life. We herewith proposed the formulation development of STAT6 siRNA (S6S) nanotherapeutic agent by encapsulating them within gelatin nanocarriers (GNC). The prepared nanoformulation was characterized for size, charge, loading efficiency, release kinetics, stability, cytotoxicity, and gene silencing assay. The stability of S6S-GNC was also assessed under conditions of varying pH, serum level, and using electrophoretic assays. *In vitro* cytotoxicity performance was evaluated in human adenocarcinoma A549 cells following MTT assay. The developed formulation resulted in an average particle size, surface charge, and encapsulation efficiency as 70 ± 6.5 nm, +10 ± 1.5 mV, and 85 ± 4.0%, respectively. S6S-GNC showed an insignificant (*P* < 0.05) change in the size and charge in the presence of buffer solutions (pH 6.4 to 8.4) and FBS (10% v/v). A549 cells were treated with native S6S, S6S-lipofectamine, placebo-GNC, and S6S-GNC using untreated cells as a control. It was observed that cell viability was decreased significantly with S6S-GNC by 55 ± 4.1% (*P* < 0.001) compared to native S6S (2.0 ± 0.55%) and S6S-lipofectamine complex (40 ± 3.1%). This investigation infers that gelatin polymer-based nanocarriers are a robust, stable, and biocompatible strategy for the delivery of siRNA.

## 1. Introduction

RNAi is a naturally occurring gene silencing process that has the advantages of a high degree of specificity and the potential to silence genes of interest [1]. Small-interfering RNAs (siRNA) are synthetic double-stranded RNA (dsRNA) of 21–23 base pairs that can be designed to suppress target sequences, in a process known as posttranscriptional gene silencing. In order to exert the therapeutic effect, the siRNA must be incorporated into the multiprotein RNA-induced silencing complex (RISC) [2]. The siRNAs, as a class of therapeutic agents, are capable of efficient knockdown of targeted genes and may have a more rapid bench-to-bedside development compared to other conventional anticancer therapies and have potential in the treatment of other gene-related disease states [3]. 

The signal transducer and activator of transcription 6 (STAT6) is one of the most prominent transcription factors that regulate gene expression in response to extracellular polypeptides that lead to cellular proliferation, differentiation, and apoptosis [4]. STAT6 is a member of a transcription factor family that is present in the latent form within the cytoplasm cells and is promptly phosphorylated by the Janus kinase (JAK) family of the tyrosine kinases following the interaction of IL4 and IL13 with the IL4 receptor alpha (IL4R*α*) and IL13 receptor alpha (IL13R*α*), respectively [4]. When activated by Janus kinases, STAT6 translocates to the nucleus where it may regulate cytokine-induced gene expression. The phosphorylated STAT6 is required for responsiveness to IL-4 and IL-13 [5]. Protein phosphatase 2A is also involved in the regulation of IL-4-mediated STAT6 signaling [6]. STAT6 has often been associated with asthma and allergic inflammation; however, it has been shown to have a role in other disease states [7, 8]. STAT6 knockdown has been shown to inhibit proliferation and induce apoptosis in HT-29 colon cancer cells [9]. It has also been reported that STAT6 is a survival factor in human prostate [10] and unphosphorylated STAT6 increases the expression of COX-2, thereby protecting nonsmall cell lung cancer (NSCLC) against apoptosis [11, 12]. Dubey et al. found that STAT6 silencing in NCI-H460 lung cancer epithelial cells leads to an increase in cholesterol production and confirmed the antiapoptotic effects of STAT6 [13]. The STAT6 transcription activator is activated by an IL4-dependent pathway and is found to upregulate the expression of BCL2L1/BCL-X(L), which is directly responsible for the antiapoptotic effect of IL4 [13, 14]. Unfortunately, currently no STAT6 inhibitors have been approved by the FDA, and those within experimental studies (like leflunomide, pitrakinra, and salicylates) have poor clinical efficacy, pharmacokinetic properties, and adverse effects [15, 16], and this highlights the need to develop alternative STAT6 targeting approach.

**Figure 1:**
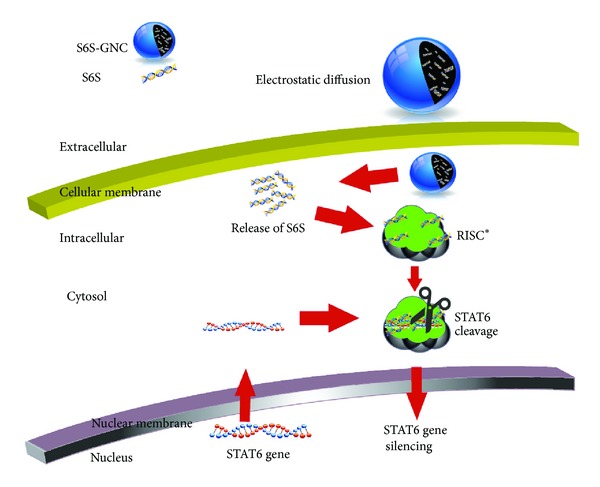
Cellular uptake and intracellular mechanism of action (MOA) of targeted S6S-GNC.

The clinical utility of siRNA has been limited to its inherent properties; for example, naked siRNA is prone to degradation and has a shorter plasma half-life, rapid renal clearance, and limited permeability across cell membranes [17]. A variety of nanocarrier (NC) systems in early cancer therapeutic clinical results showed enhanced efficacy and reduced side effects [18–27]. Cationic lipid-based systems have emerged as the most attractive for siRNA delivery; however, the use is limited due to poor transfection efficiency and toxicity [28]. Natural polymer-based delivery systems are biocompatible and biodegradable with high physiological tolerance and low immunogenicity [29–35]. To circumvent this difficulty with siRNA delivery, we have formulated S6S as a nanotherapeutic agent by encapsulating siRNA within a gelatin nanocarrier (GNC). Gelatin is a FDA approved natural biopolymer, which is inexpensive, biodegradable, and nontoxic, has low antigenicity, and is easily modified [36]. We hypothesized that the development of FDA approved gelatin-based nanotherapeutics of S6S will provide biostability and will deliver the S6S to the tumor cells and thereby will exert a significant anticancer activity. The mechanism of S6S cellular delivery will be achieved via electrostatic diffusion as illustrated in Figure 1. The core objective of this investigation was to develop and evaluate the biocompatible S6S nanoformulation that will enable effective intratumoral delivery.

## 2. Materials and Methods

### 2.1. Materials

Gelatin (type A; 175 g Bloom Strength; with isoelectric point of 8-9, and average molecular weight of 40–50 kDa; GELITA, USA) was graciously provided as a gift from the manufacturer. Glutaraldehyde (GTA) was purchased from Alfa Aesar (Heysham, Lancaster) as a 25% aqueous solution. Genipin (GEN) was kindly provided as a gift sample from Wilshire Technologies, Inc. (Princeton, NJ, USA). Acetaminophen (APAP) was purchased from Sigma Aldrich. STAT6 siRNA was purchased from Santa Cruz Biotechnology, Inc. (Santa Cruz, CA, USA). Ethanol, dimethyl sulfoxide (DMSO), 3-[4,5-dimethylthiazol-2-yl]-2,5-diphenyl tetrazolium bromide (MTT), and lactose monohydrate were purchased from VWR International (Radnor, PA, USA). Spectra/Por Dialysis membranes (MWCO 25 kDa and 100 kDa) were obtained from Spectrum Laboratories, Inc. (Rancho Dominguez, CA, USA). Distilled deionized and 0.22 *μ*m filtered sterile water were used throughout the experiments. All other chemicals were either reagent or tissue culture grade.

#### 2.1.1. Cell Culture

The A549 (human lung adenocarcinoma epithelial cell line, CCL-185, ATCC, Manassas, VA) cells were grown as monolayers in 75 cm^2^ tissue culture flasks (Greiner Bio-one, Monroe, NC, USA) at 37°C under 5% CO_2_ in F12-K supplemented medium (Life Technologies, Grand Island, NY, USA) with 10% v/v fetal bovine serum (FBS) and an antibiotic antimycotic solution of penicillin (5000 U/mL), streptomycin (0.1 mg/mL), and neomycin (0.2 mg/mL) (PSN). Cell culture media and PSN stock solutions were purchased from Cellgro (Herndon, VA, USA). Heat inactivated FBS was purchased from Atlanta Biologicals (Lawrenceville, GA, USA).

### 2.2. Methods

#### 2.2.1. Selection of Gelatin Molecular Weight Fraction by Controlled Desolvation

The GNCs formed from the whole gelatin fraction were prepared by one step desolvation technique. Briefly, a 1% w/v gelatin type “A” solution was prepared by dissolving gelatin in distilled deionized H_2_O at 50°C under gentle stirring at 400 rpm. When the gelatin solution became homogeneous and transparent, the temperature of the solution was reduced to 35°C and 19.98 mg acetaminophen (model drug engaged to optimize formulation conditions), added, and dissolved. Then, the desolvation step was accomplished, wherein 80% (v/v) ethanol was added at a rate of 1 mL/min under constant stirring at 600 rpm. Following this, 150 *μ*L 10% GTA was added at a rate of 0.2 mL/min to crosslink the nanocarriers. The formulation was stirred at a rate of 600 rpm for another 55 min, and the stir rate was reduced to 200 rpm until ethanol completely evaporated (process required approximately 12 hr).

The high molecular weight (HMW) fraction was prepared by the classical 2-step desolvation technique, where 5% (w/v) gelatin type “A” was first desolvated with an equal volume of acetone for 12 minutes under gentle stirring. After 12 minutes, the supernatant that contained the low molecular weight (LMW) gelatin fraction, water, and acetone was decanted and discarded. The HMW fraction sediment was allowed to dry and underwent mass reconciliation. The HMW gelatin was redissolved in distilled deionized H_2_O 1% (w/v) solution at 50°C under gentle stirring. When the gelatin solution became homogeneous and transparent, the temperature of the solution was reduced to 35°C and 19.80 mg acetaminophen was added and dissolved. Then, a second desolvation step commenced, where 80% v/v pure ethanol was added dropwise at a rate of 1 mL/min under a constant stirring rate of 600 rpm. Five minutes after the ethanol addition ended, 150 *μ*L 10% GTA was added drop-wise at a rate of 0.2 mL/min to crosslink the gelatin and therefore harden the nanocarriers. The formulation was stirred at a rate of 600 rpm for another 55 min, and then 5 mL distilled deionized H_2_O was added and the stir rate was reduced to 200 rpm until ethanol completely evaporated.

The MMW fraction was prepared by a modified 2-step desolvation technique, where 5% w/v gelatin type “A” was first desolvated with an equal volume of acetone for 5 seconds, quickly decanted into another beaker, and then allowed to desolvate for another 12 minutes where the LMW fraction was decanted and discarded. The first contains HMW fraction, while the LMW gelatin in water and acetone supernatant was discarded. The MWW fraction sediment was allowed to dry and underwent mass reconciliation.

The MMW gelatin was redissolved in distilled deionized H_2_O to make a 1% w/v solution at 50°C under gentle stirring at 400 rpm. When the gelatin solution became homogeneous and transparent, the temperature of the solution was reduced to 35°C, and 22.92 mg acetaminophen was added and dissolved. Then, a second desolvation step commenced, where 80% pure ethanol was added dropwise at a rate of 1 mL/min under constant stirring at 600 rpm. Five minutes after the ethanol addition ended, 150 *μ*L of 10% GTA was added dropwise at a rate of 0.2 mL/min to crosslink gelatin and therefore harden the nanocarriers. The formulation is stirred at a rate of 600 rpm for another 55 min, and then 5 mL distilled deionized H_2_O was added, and the stir rate was reduced to 200 rpm until ethanol completely evaporated.

The whole, HMW, and MMW gelatin fractions were compared for their resultant nanocarrier particle size, polydispersity index, and entrapment efficiency (EE%).

#### 2.2.2. Formulation and Optimization of Gelatin Nanocarrier Using Taguchi Orthogonal Array Design

**Figure 2:**
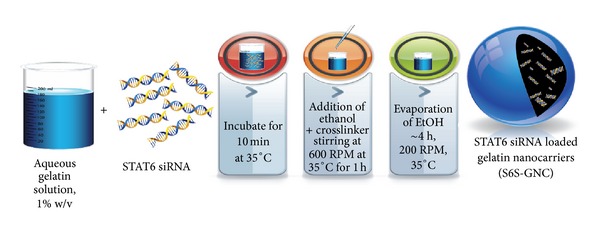
Preparation of S6S-GNC. The 1% w/v aqueous gelatin solution is incubated with the STAT6 siRNA for 10 min at 35°C, and then ethanol and crosslinker are added dropwise at a stirring rate of 600 rpm at 35°C for 1 hr, at which point the stirring rate is reduced to 200 rpm. After approximately 4 h, the ethanol is completely evaporated, and STAT6 siRNA loaded gelatin nanocarriers remain in a colloidal suspension in water or PBS pH 7.4. The resultant nanoparticles were collected by centrifugation and resuspended for subsequent characterization or lyophilization in the presence of 1% w/w lactose monohydrate.

Type A gelatin-based nanocarriers were prepared using the 2-step desolvation technique with slight modifications (Figure 2) [37, 38]. The formulated GNC was crosslinked with more biocompatible crosslinker, GEN, as against predominantly employed GTA crosslinker [39, 40]. Briefly, GNC formulations were optimized using a Taguchi orthogonal array design with the independent variables being stir rate, ethanol volume, and GEN concentration with particle size being the dependent variable. For this investigation, APAP was used as a model drug to set formulation parameters. This optimized formula was used to prepare S6S loaded gelatin nanocarriers as briefed in the following sections of the paper.

#### 2.2.3. Preparation of S6S Loaded Gelatin Nanocarriers (S6S-GNC)

**Figure 3:**
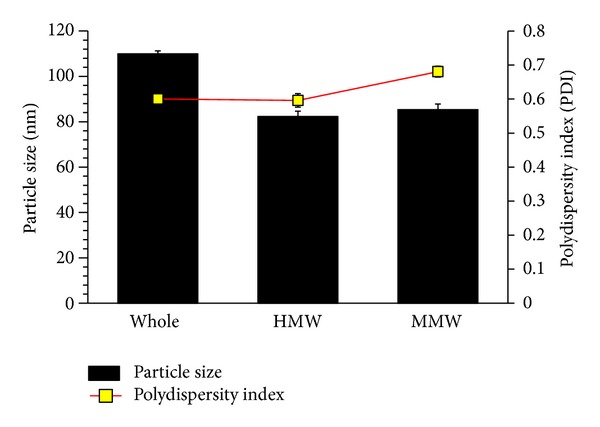
Particle size analysis report for GNC formulated at 600 rpm stir rate (magnetic stir bar method). Data pertains to nanocarrier formulation at stir rates of 300 rpm and 700 rpm which were not included because the particle sizes obtained therein were outside acceptance range. The bars, dots, and error bars represent the mean ± standard deviation (*n* = 3).

S6S-GNC was formulated by employing the optimized 2-step desolvation methodology (Figure 2) with slight modifications [37, 40]. HMW gelatin fraction (Figure 3) that generated small sized nanocarrier was engaged for formulation development. One key amendment was made in relation to desolvating solvent, wherein diluted ethanol was employed in our method as compared to 100% ethanol in reported methods of gelatin nanoparticle preparation [37, 38]. It was anticipated that the use of a diluted ethanol solution (aqueous) will generate a milder environment for desolvation and hence lessen the chance to form larger, nonuniformly packed gelatin nanocarriers during the preparation stage. Briefly, 9 mL of 9 : 1 (v/v) ethanol to water solution was added to 10 mL (total aqueous ethanol, 90% v/v) drop-wise at a temperature of 35°C at an injection speed of 1 mL/min). After ethanol addition, 200 *μ*L of 5 mg/mL GEN was added to dropwise to crosslink the formed nanocarriers. After 1 hr, the temperature and stir rate were reduced to 30°C and 200 rpm, respectively, in order to avoid thermal, as well as mechanical stress-induced aggregation or agglomeration. The resultant nanocarriers were purified by three cycles of centrifugation at 10,000 ×g for 30 min followed by dispersion of the pellet in PBS pH 7.4 to the original volume.

#### 2.2.4. Particle Size, Zeta Potential Measurement

The particle size of the prepared S6S-GNC was determined by dynamic light scattering using a NICOMP ZLS 380 analyzer (PSS-NICOMP, Santa Barbara, USA) [41, 42]. The particle size of the S6S-GNC was assessed by dispersion in phosphate-buffered saline (PBS) pH 7.4. The zeta potential of the S6S-GNC was assessed by dispersion in distilled deionized sterile water. The zeta (*ζ*) potential was calculated by Smoluchowski's equation from the electrophoretic mobility of the S6S-GNC at 25°C. All measurements were recorded in triplicate. The number of GNC per mL of suspension will be calculated using the size of the GNC determined as described previously [43] using the following formula. *N* = *φ*/[4/3*π*(*d*/2)3], where *N* is the number of GNC/volume, *φ* is the volume fraction of particles determined by viscosity, 4/3*π*(*d*/2)3 is the average volume of a GNC, and *d* is the volume-weighed diameter determined by light scattering.

#### 2.2.5. Determination of Entrapment Efficiency (%)

The entrapment efficiency was determined by employing Vivaspin500 ultracentrifuge filters (MWCO100 kDa, Viva Products, Inc., Littleton, MA, USA) using UV spectrophotometry to quantify the free siRNA in a sample. Briefly, S6S-GNC formulation was placed on the top of the Vivaspin filter membrane and centrifuged at 16,200 ×g for 10 min. The aqueous filtrate was then subjected to UV spectrophotometric analysis to determine the free S6S content using a BioSpek-nano Micro-volume UV-Vis Spectrophotometer (Shimadzu, Columbia, MD, USA). Sample sizes of 2 *μ*L were loaded onto the sample mount and then analyzed using a pathlength of 0.7 mm. The entrapment of S6S within the developed formulations was calculated using the following equation:

(1) Entrapment  Efficiency  (EE%)  =Total  S6S  added  (nM)−Free  S6S  (nM)Total  S6S  added  (nM)×100.

#### 2.2.6. *In Vitro* Release Profile of S6S from S6S-GNC

S6S release from S6S-GNC formulation was assessed under physiological pH employing phosphate-buffered saline (PBS; pH 7.4) as release milieu. Briefly, two mL of S6S-GNC formulation was placed inside a dialysis bag (MWCO 100 kDa, Fisher Scientific, USA). The membrane bags were placed in 50 mL of PBS pH 7.4 under constant agitation condition (300 rpm) at 37°C. At predetermined time intervals (0.5, 1, 2, 3, 4, 5, 6, 7, 24, 48, 72 hr), 0.5 mL of dissolution medium was collected, and equal volume of fresh dissolution medium was replaced to simulate perfect sink conditions. The samples were analyzed at each time interval using the S6S from the developed formulations.

#### 2.2.7. Serum and pH Stability

Stability of S6S-GNC under conditions of varying pH and serum level was also assessed to investigate the stability of developed formulation under different environments [44]. The S6S-GNC was incubated in PBS pH 6.4, 7.4, and 8.4 for 1 hr to assess the influence of pH on the surface charge and size of the nanoformulations. Furthermore, to develop proof that the GNC will eventually prevent *in vivo* degradation of S6S, stability studies were also performed under the presence of 10% v/v FBS at pH 7.4.

#### 2.2.8. S6S Stability Study: Agarose Gel Electrophoretic Mobility Assay

Stability of encapsulated S6S was also assessed by agarose gel electrophoresis as described previously [45], with slight modifications. The assay was performed to examine the stability of encapsulated siRNA in its loaded as well as solution form. To mimic siRNA exposure under *in vivo* condition, stability was also assessed in the presence of RNAse, an enzyme that degrades siRNA [46, 47]. The 1% (w/v) agarose gel was electrophoresed at a constant voltage of 70 V until the bromophenol blue marker bands were well separated.

#### 2.2.9. *In Vitro* Cytotoxicity of S6S-GNC

The effect of S6S-GNC on viability of A549 cell lines was measured using established MTT assay protocol [48]. Briefly, A549 cells were seeded into 96-well plates at a density of 10^4^ cells/well and incubated overnight. After this, cells were treated with S6S, S6S lipofectamine complex, placebo-GNC, and S6S loaded GNCs for 24 and 48 hr. The 96-well plates were incubated at 37 ± 0.2°C, and the cell viability was measured using MTT assay [48]. The untreated cells were used as control.

#### 2.2.10. Cell Internalization Assay

The cell internalization was evaluated by treating Nile red dye loaded GNC in A549 cells [49]. Briefly, A549 cells were seeded in 24-well plates (25,000/well) and were incubated with Nile red loaded GNC followed by imaging of cells at 0, 0.25, and 1 hr. The cellular uptake of the S6S-GNC A549 lung cancer cells was investigated with fluorescence microscopy using a Zeiss Axiovert 40 CFL inverted microscope with an appropriate filter set (Carl Zeiss Microscopy, LLC, USA). The fluorescent light source was an Exfo X-Cite series 120 (Lumen Dynamics Group, Inc., Mississauga, Ontario, Canada). 

#### 2.2.11. Measurement of STAT6 Protein by Western Blot

In order to assess the efficiency of STAT6 silencing by various formulations under investigation, expression levels of STAT6 protein were monitored following nanoformulation treatment with appropriate controls. Briefly, untreated control, S6S + lipofectamine complex, and S6S-GNC-treated A549 cells were lysed using RIPA buffer, and sodium dodecyl sulfate-polyacrylamide gel electrophoresis (SDS-PAGE) was performed as per previously described [50, 51]. The SDS-PAGE gel was electrophoresed at 45 V for 30 min and then 120 V until the bromophenol blue markers were well separated. Human STAT6 and *β*-Actin proteins (Cell Signaling, USA) were detected using rabbit polyclonal primary antibodies (Santa Cruz Biotechnology, Inc., USA). The STAT6 antibody utilized was capable of detecting endogenous levels of total STAT6 protein. The primary antibodies were tagged with secondary anti-rabbit IgG antibody horseradish peroxidase- (HRP-) linked antibody. The affinity purified goat anti-rabbit IgG (H&L) antibody was conjugated to horseradish peroxidase by the supplier/manufacturer for use as a secondary antibody in chemiluminescent western blotting applications. Proteins were visualized using Luminol Reagent (Bio-Rad, USA). 

### 2.3. Statistical Analysis

The experiments were conducted in triplicate with data reported as mean ± standard deviation. Experimental statistics were analyzed using Minitab 16 Statistical Software (State College, PA, USA). The significance level was set at *P* < 0.05.

## 3. Results and Discussion

According to a recent report by American Cancer Society, cancer is a leading cause of death in the United States, and by end of year 2013, approximately half a million Americans are anticipated to succumb to cancer [52]. Current lung cancer treatment modalities include surgery, chemotherapy, radiation therapy, and several new investigational approaches that are now being tested including photodynamic therapy, immunotherapy, and gene therapy [53–55]. However, surgery and radiotherapy are not viable in most patients, while chemotherapy results in low response rates with adverse side effects [56, 57]. Hence, the development of newer and more effective pharmacological interventions is needed for the treatment of cancer.

The aim of this this investigation was to provide proof of concept that gelatin polymer (FDA approved polymer) based nanocarrier formulations of S6S will provide alternate mode to attain therapeutic benefit of siRNA in cancer therapy [58].

Gelatin is a biodegradable/biocompatible polymer approved by FDA for I.V. administration. Gelatin-based nanoparticles represent an attractive strategy, since a significant amount of bioactive can be incorporated into the protein-based nanoparticle matrix [59, 60]. Among the two subtypes of gelatin (Type-A and B), type A gelatin is positively charged at about pH 5; hence, type A gelatin was used to avail pH-dependent protonation efficiency of gelatin [61, 62]. It should be noted that type B gelatin has been previously used for siRNA delivery [59]; however, reports on comparative grounds between type A and type B gelatin clearly infer type A gelatin to be fitting for siRNA delivery. The gelatin type A has net positive charge that allows the efficient encapsulation of positively charged siRNAs [63–65]. Therefore, gelatin type A has been selected to formulate the S6S encapsulated nanocarriers. 

For the preparation of GNC's, a two-step desolvation technique was utilized, wherein in first step, the gelatin type A was fractionated to remove the LMW fraction using acetone as a desolvating agent, and then the second step was performed to form the nanocarriers [37, 40]. A schematic outline of formulation process has been illustrated in Figure 2. We have utilized the electrostatic interactions between the negatively charged siRNA and positive charge gelatin (type A, IEP ~8-9) to formulate the S6S encapsulated GNCs. The formulation strategy followed by us differs from the previously described methods, for example, by Kommareddy and Amiji and Lemieux et al., where neutral or negative charged noncondensing lipids or polymers and the negatively charged oligonucleotide payload are encapsulated by the physical entanglement of nucleic acid constructs within the matrix or through hydrogen bonds between the polymer and nucleic acid bases [66, 67]. Electrostatic interaction as a means of oligonucleotide or siRNA loading has been used successfully in previous studies [62, 68]; however, optimization of the formulation parameters has not been accomplished to reduce the particle size to desired range for enhanced cancer targeting (size < 100 nm) [69, 70].

The effect of varying gelatin molecular weight on formulation of GNC was also studied by Coester et al. in 2000, wherein molecular weight of gelatin was reported to be greatly influencing the stability as well as particle size of the developed gelatin nanocarriers [37]. In view of studying the influence of various molecular weight fractions on formulation of GNCs, we have performed a systematic studies in this investigation. Our investigation on varying molecular weight fractions of gelatin illustrated that the HMW fraction had apparent advantages over the whole gelatin in respect to producing lower particle size of the resultant nanocarriers, which is in agreement with previously reported findings [37, 40]. Since HMW gelatin fraction produced smaller particle sized nanoparticles, it was anticipated that the medium molecular weight (MMW) fraction might produce further lower particle size. Typically, in nanocarrier formulation, the LMW polymers lead to formation of smaller sized nanocarriers [71, 72]. The GNC formulated with MMW fraction resulted in comparatively smaller sized nanocarrier as compared to HMW, but the variance, or the polydispersity index (PDI), was significantly higher in case of MMW (Figure 3). However, from the outcomes of our investigation, it can be evinced that there is nonsignificant difference between the HMW and MMW gelatin fractions based nanocarriers formulation (*P* > 0.005; Figure 3). This larger PDI was unexpected since the LMW fraction-based nanocarriers were anticipated to be capable of producing smaller sized particles. It may be possible that the unique combination of gelatin molecular weights remained after desolvation process might had allowed tighter packing in the spherical gelatin nanocarrier, similar to the tighter molecule packing between two different molecular weight fractions in cocrystals compared to pure crystals [73]. Conclusively, as shown in Figure 3, the HMW fraction generated more robust nanocarriers with a lower PDI. Therefore, we have selected the HMW fraction for further development of S6S-GNC formulation.

**Figure 4:**
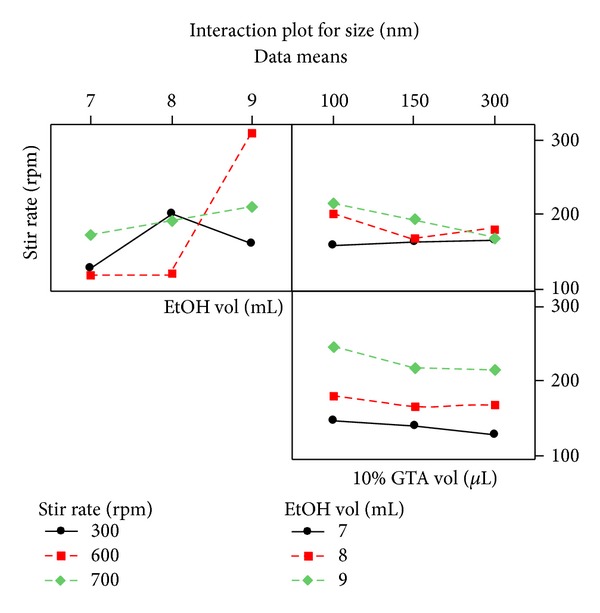
Interaction plot for the dependent variable particle size in the Taguchi orthogonal array experimental design for the formulation development of GNC.

GNC formulations were optimized using a 3^3^ Taguchi orthogonal array design with the independent variables being stirring rate, ethanol volume, and GEN concentration and the dependent variable of particle size (Table 1). Taguchi orthogonal array design has been used extensively in the literature to evaluate the critical factors and develop the optimal formulation by reducing the number of experiments by using the orthogonal array design. Thus, this approach reduces cost and time associated with formulation optimization [74, 75]. In this investigation, we have employed Taguchi orthogonal array design to identify the relative significance of numerous variables and their interactions [76–78]. For the systematic optimization studies, APAP was employed as a model drug based on the hydrophilic nature and negative charge (pKa isoelectric point 9.38) which resembles siRNA properties [79–81]. The outcomes of these investigations are presented in Figure 4. The optimized parameters were found to be 600 rpm stirring rate, 7 mL of ethanol added as desolvating agent, and 300 *μ*L of 10% GTA. The stir rates of 300 and 600 rpm lead to similar particle size means. Stir rate of 700 rpm generated much higher particle size means compared to the GNC prepared at 300 and 600 rpm. The crosslinker concentration in interaction with stir rate did not influence the particle size. The ethanol volume added had great influence on the particle size means with interaction with the crosslinker concentration. The formula optimized using APAP as a model drug was then engaged to formulate S6S-GNC with slight modifications. Since the optimized ethanol percent volume added to the gelatin solution was 80% v/v, a 9 : 1 (v/v) ethanol to water solution was prepared, and the final percent volume of total aqueous ethanol desolvating agent to be added was increased to 90% (v/v).

We have also utilized a modified two-step desolvation technique to prepare the GNC as a colloidal delivery system, and the key factors effecting formulation of GNC were considered in the preparation of the nanoformulation. Particle size is a highly influential dependent variable that influences the cellular uptake of nanoparticles and the tissue and organ distribution of nanoparticles [82]. The nanocarriers with size of <100 nm were shown an improved efficacy due to the associated enhanced permeation and retention effects due to leaky tumor vasculature and improved pharmacokinetics [83].

**Figure 5:**
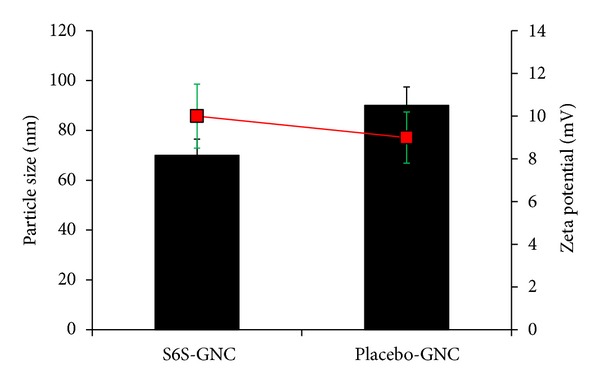
Particle size and zeta potential of the S6S-GNC batches and placebo-GNC. The bars represent particle size (nm), the square markers represent the zeta potential (mV), and the error bars represent standard deviation.

Also, body distribution studies have shown that nanoparticles >230 nm will accumulate in the spleen because of the capillary diameter within this organ [84]. Hence, optimization of gelatin nanoparticles should be performed critically to achieve the desired properties and therapeutic effects. As shown in Figure 5, the particle size and surface charge of the optimized S6S-GNC formulation were observed to be 69.6 ± 6.5 nm and +10 ± 0.56 mV, respectively. Other studies that aimed to formulate gelatin nanoparticles have shown the particle size of >100 nm [37, 68, 85]. The entrapment efficiency of the S6S-GNC formulation was found to be 85 ± 2.87%. The developed formulation contained 10,000 GNC per mL [42, 43, 86]. The S6S-GNCs were found to be within the desired formulation characteristics range (<100 nm particle size, >+5 mV surface charge, and >80% EE%).

**Figure 6:**
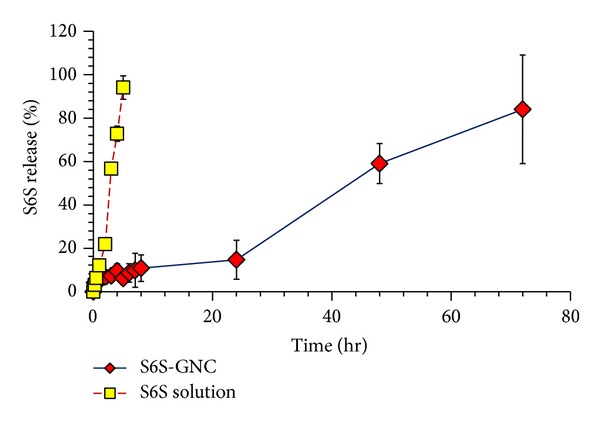
*In vitro* STAT6 siRNA release profile for the S6S-GNC formulation compared to the STAT6 siRNA solution. Lyophilized formulation was resuspended in PBS pH 7.4 and filled inside dialysis membrane bags with MWCO of 300 kDa (Sigma, USA). The membrane bags were placed in 50 mL of PBS medium maintained at a temperature of 37 ± 2°C with continuous gentle stirring at 300 rpm on a magnetic heating and stirring plate. At specific time intervals, 0.5 mL aliquots of dissolution medium were withdrawn and analyzed using a Biospek UV spectrophotometer. Results are represented as mean ± standard deviations (where *n* = 3).

The *in vitro* profile release of S6S from the S6S-GNC formulations as compared to plain S6S solution in PBS media is shown in Figure 6. Developed S6S-GNC formulation showed sustained release of encapsulated S6S, inferring the efficient cargo retentive property of developed formulation (Figure 6). The S6S-GNC showed <15% S6S release at 24 hr, ~50% release at 48 hr, and ~84% release at 72 hr time points. Burst release of approximately 5.0% was observed upon incubation of the nanoformulation to the PBS pH 7.4 inferring that only small fraction of loaded S6S is associated with the surface of the GNC, while the majority of S6S is within the gelatin matrix of formed GNCs [87]. A sustained release of loaded bioactive from gelatin nanoparticles was also observed by earlier investigators, and our results are in agreement with the existing reports [69, 88].

**Figure 7:**
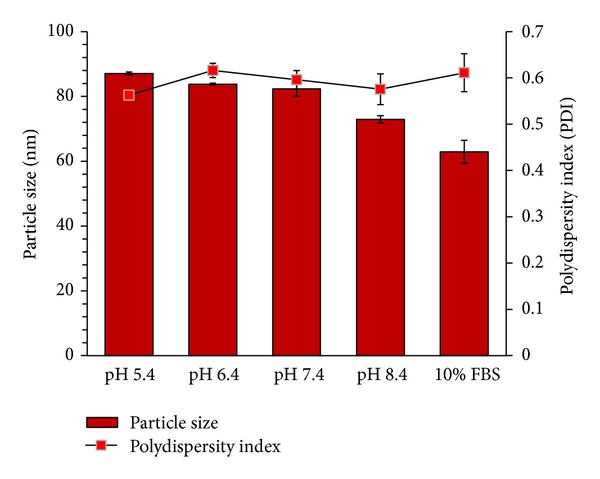
Stability of S6S-GNC formulation. Outcome is as expressed by size (nm) and zeta potential (mV) under the influence of varying pH between 5.4 and 8.4 and 10% v/v FBS at physiological pH 7.4 to mimic the serum found in human blood. Results are represented as mean ± SD (*n* = 3).

It was widely reported that encapsulation of bioactive agents in the nanoparticles significantly ameliorates as well as prevent degradation of loaded bioactivities [70, 89]. Hence, in order to generate a proof behind our hypothesis that GNC will eventually prevent *in vivo* degradation of S6S, stability studies were performed under presence of buffer solutions and 10% v/v fetal bovine serum. S6S-GNC showed a nonsignificant (*P* > 0.05) change in the size and charge in the presence of buffer solutions in the pH range of 6.4 to 8.4 (Figure 7). Similarly, a nonsignificant (*P* > 0.05) change in the size and charge was observed in presence of FBS/PBS. The degree of GNC and serum protein interaction depends highly on the size and charge characteristics of the GNC [90]. Reticuloendothelial system (RES) uptake in the liver or spleen occurs due to the protein absorption, which then leads to opsonization and subsequent phagocytosis by macrophages [91]. Our developed S6S-GNC formulation was stable in terms of size under the physiological conditions. 

**Figure 8:**
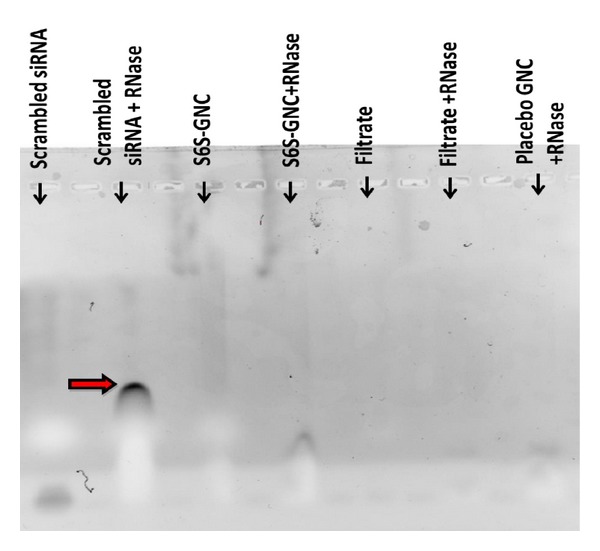
Agarose gel electrophoretic mobility shift assay. The scrambled siRNA control, scrambled siRNA treated with RNAse control, S6S-GNC, S6S-GNC treated with RNAse, filtrate, filtrate treated with RNAse, and the placebo-GNC treated with RNAse were loaded onto a 1% w/v agarose gel and electrophoresed at a constant voltage of 70 V until the bromophenol blue marker bands were well separated. This study was performed to examine the stability of the encapsulated siRNA due to preparation conditions, as well as the stability in the presence of RNAse. The arrow head indicates the distance traveled by the cleaved siRNA fragments.

Additionally, agarose gel electrophoretic mobility shift assay was also performed to assess the stability of entrapped siRNA during formulation conditions and exposure to RNAse.  Figure 8 shows the electrophoretic mobility pattern of siRNA from the S6S-GNC compared to that of appropriate controls. The bands indicated that the naked S6S had been enzymatically degraded by the RNAse and therefore moved through the 1% agarose gel more rapidly compared to that of S6S entrapped with the GNC matrix (Figure 8). Intact super coiled RNA was recovered from the native scrambled siRNA-loaded lane. The treatment with RNAse only enzymatically degraded the S6S alone, and this effect was not noted in case of S6S-GNC. The S6S-GNC was found to be protecting the S6S from RNAse degradation. The filtrate was found to be containing only a minute proportion of added S6S, and this suggests the entrapment of S6S within the matrix of gelatin nanocarrier. The same fact can be again correlated with the observed high S6S entrapment efficiency of ~85%. In further experiments, S6S-GNCs were intentionally allowed to release their contents following the treatment with protease to confirm that the S6S was entrapped within the GNC. Intact super coiled RNA was recovered from GNC formulation treated with protease. In agreement with our findings, Kriegal and Amiji showed protection of encapsulated siRNA from degradation using gelatin nanoparticles [45]. Our results demonstrate the formulation of a stable and functional siRNA loaded GNC (S6S-GNC) that can protect the siRNA from nucleases during systemic circulation.

**Figure 9:**
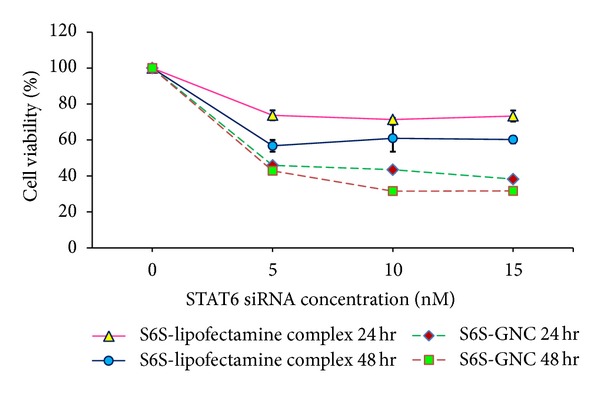
Cytotoxicity of the developed S6S-GNC compared to placebo, native S6S and S6S with lipofectamine on A549 lung cancer cells. The graph shows the % cell viability observed after 24 and 48 hr following treatment. Cell viability was performed using 5 × 10^3^ A549 (human adenocarcinoma cell line) cells in F12-K medium supplemented with 10% FBS and an antibiotic antimycotic solution of penicillin (5000 U/mL), streptomycin (0.1 mg/mL), and neomycin (0.2 mg/mL) (PSN). Cell incubation was conducted within a humidified atmosphere of 5% CO_2_ at a temperature of 37 ± 0.5°C. The formulation and S6S lipofectamine complexes were applied as freshly prepared solutions between 0 and 15 nM concentrations. The absorbance of the formazan crystals dissolved in DMSO was read at 540 nm on a Biospek Synergy H1 plate reader. Values are represented as mean ± SD (where *n* = 3).

After physiochemical characterization of GNC formulation, we have evaluated the *in vitro* cytotoxicity of the S6S loaded formulation which was evaluated against model A549 lung cancer cells following established MTT cytotoxicity assay [18, 23, 24]. Human adenocarcinoma A549 slow growing cells were selected for this investigation based as reported expression of STAT6 protein [13]. We observed that the percent cell kill was increased significantly (*P* < 0.001) with S6S-GNC (55 ± 4.1%) compared to native S6S (2 ± 0.55%) and S6S with lipofectamine (40 ± 3.1%) (Figure 9). In agreement with our findings, Shah et al. found that the HIF-1*α* siRNA loaded gelatin or PEG-modified gelatin nanoparticle treatment of the known HIF-1*α* overexpressing cell lines, SKOV3 and MDA-MB-231 cells, significantly inhibited the expression of HIF-1*α* compared to that of naïve siRNA, therefore reversing the aggressive phenotype of these tumors [59, 92, 93]. Placebo-GNC treatment showed >97% viability of cells demonstrating nontoxicity and safety of gelatin used in the GNC formulation (Figure 9).

**Figure 10:**
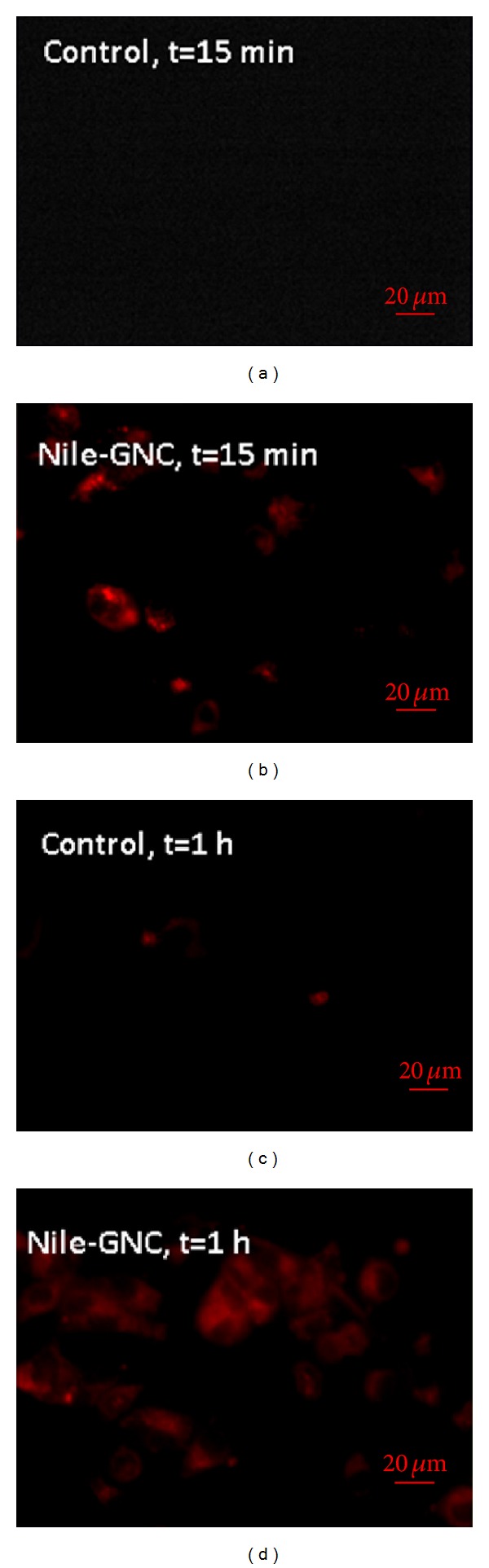
Fluorescence images of control and Nile red loaded GNC (Nile-GNC) taken after 15 min and 1 h of treatment in A549 cells (at 40x magnification). For this assay, lung cancer A549 cells (5 × 10^4^) were seeded on 24 well plates and incubated at 37 ± 0.5°C under 5% CO_2_ for 24 h. The cells were treated with the Nile red solution or Nile red loaded formulations for 15 min and then treated for an addition of 45 min for a total treatment time of 1 hr. After 15 min, media were removed, and the resulting cells were washed with PBS.

The cell internalization of nanoparticles plays an important role in eliciting therapeutic effect [94]. Therefore, we have performed cellular uptake studies employing Nile Red loaded GNC instead of cell binding studies which help us to understand the uptake of nanocarriers within the cells [95]. Nile red is capable of fluorescent staining of intracellular lipid droplets that excites and emits at wavelengths similar to red fluorescent protein at approximately 485 nm and 525 nm, respectively [96].  Figure 10 shows the fluorescence images of the control Nile-Red solution and the Nile-Red loaded GNC (Nile-GNC) taken after 15 min and 1 hr of treatment in A549 cells at 40x magnification. The Nile red loaded GNC clearly outperformed the Nile red solution at 15 min and 1 hr time points as shown by the apparent increase in the fluorescence associated with GNC (Figure 10). The cell internalization studies of Nile red loaded GNC formulations showed that the developed GNC formulation gets rapidly internalized within cells compared to free dye controls.

**Figure 11:**
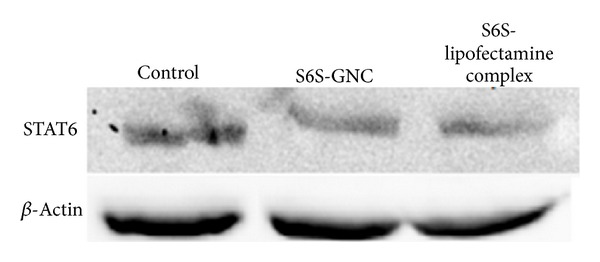
Measurement of STAT6 protein expression by western blot. The effect of STAT6 siRNA-GNC on the expression of STAT6 in A549-treated lung cancer cells was shown. A549 cells were preincubated with S6S-GNC and S6S-lipofectamine complex and without any treatment (control). The cells were lysed, and STAT6 protein expression was analyzed by western blot of whole cell lysates. The *β*-Actin expression was analyzed as a loading control.

STAT6 protein is involved in tumor progression and resistance and was reported to be activated by phosphorylation; therefore, downstream expression of pSTAT6, in addition to other downstream proteins, IFN*γ*, TGF-*β*, Foxp3, IgE, and GATA3, plays important role in the tumor progression due to the resultant favoring of Th2 differentiation, cell cycle promotion, and antiapoptotoc and prometastatic properties [97]. The phosphorylation of STAT6 to pSTAT6 leads to increased expression of GATA3 and hampered expression of IFN*γ*, TGF-*β*, and Foxp3 [98]. Thus, inhibition of STAT6 using S6S-GNC will inhibit the effects of IL4 and promote upregulation of IFN*γ* and TGF-*β*/Foxp3 [99]. A western blot experiment was performed to determine the effect on STAT6 downregulation in response to the treatment with the S6S-GNC as compared to the S6S-lipofectamine complex and the negative control.  Figure 11 shows the effect of S6S-GNC on the expression of STAT6 in A549 cells. Developed S6S-GNC formulation was able to successfully downregulate the STAT6 protein expression in A549 cells thereby supporting the effectiveness of the developed formulation. In support of our results, Kriegel et al. demonstrated the downregulation of TNF-*α* using a combination of TNF-*α* and CyD1 siRNA loaded type B gelatin nanoparticles [100]. Thus, it can be stated that the strategy used in this investigation successfully leads to formulation of a safe, effective, and efficacious siRNA loaded GNC. Further formulation development of ligand anchored S6S-GNC to target S6S to cancer cell is currently under progress in our laboratory. The evaluation of S6S-GNC dose response relationships against lung cancer cells needs to be studied in order to optimize the dose required for sufficient STAT6 silencing.

## 4. Conclusion

Stable and effective S6S-GNC formulation with small particle size of <80 nm and encapsulation efficiency of >85% was successfully developed. In addition, the formulation was found to be stable in presence of buffers solutions, serum solution, and RNAase. The S6S-GNC formulation showed sustained release of S6S, which is highly desirable considering long-term effect of formulation with reduced dosing interval. S6S loaded GNC evaluated in A549 lung cancer cell line inferred significantly (*P* < 0.001) higher percent cell kill with S6S-GNC compared to that of native S6S and S6S lipofectamine. The cell internalization studies showed that the developed GNC formulation gets rapidly internalized within cells, and these results support the successful delivery of siRNA within tumor cells. Our western blot results confirmed the successful silencing of STAT6 by developed S6S-GNC formulation. The developed S6S-GNC was found to be effective in protecting S6S from degradation and able to deliver S6S within the tumor cells to exert anticancer activity. 

## Figures and Tables

**Table 1:** Taguchi orthogonal array design for the optimization of S6S-GNC using APAP as a model drug.

Factor	Levels		
	1	2	3
Stirring rate (rpm)	300	600	700
Ethanol proportions (% v/v)	70	80	90
10% GTA (*μ*L/20 mL formulation)	100	150	300
